# Palpation and Ultrasonography Reveal an Ignored Function of the Inferior Belly of Omohyoid: A Case Series and a Proof-of-Concept Study

**DOI:** 10.3390/diagnostics13183004

**Published:** 2023-09-20

**Authors:** Juan J. Canoso, José Alvarez Nemegyei, Esperanza Naredo, Jorge Murillo González, José Ramón Mérida Velasco, Cristina Hernández Díaz, Otto Olivas Vergara, José Guillermo Alvarez Acosta, José Eduardo Navarro Zarza, Robert A. Kalish

**Affiliations:** 1 Department of Medicine, ABC Medical Center, Mexico City 05348, Mexico; jcanoso@gmail.com; 2 Division of Rheumatology, Tufts University School of Medicine, Boston, MA 02111, USA; 3 Rheumatology, Star Medical Hospital, Mérida 97130, Mexico; nemegyei@hotmail.com (J.A.N.); jgaa89@gmail.com (J.G.A.A.); rkalish@tuftsmedicalcenter.org (R.A.K.); 4 Department of Rheumatology and Bone and Joint Research Unit, IIS Fundación Jiménez Díaz, Hospital Universitario Fundación Jiménez Diaz, 28040 Madrid, Spain; enaredo@ser.es (E.N.); otto.olivas@quironsalud.es (O.O.V.); 5 Department of Medicine, Universidad Autónoma de Madrid, 28049 Madrid, Spain; 6 Department of Anatomy and Embryology, Faculty of Medicine, Complutense University of Madrid, 28040 Madrid, Spain; mvlopera@med.ucm.es; 7 Department of Rheumatology, Hospital Juárez de México, Mexico City 07760, Mexico; piubella.cris@gmail.com; 8 Departamento de Medicina Interna y Reumatología, Hospital General de Chilpancingo Gro. Dr. Raymundo Abarca Alarcón, Chilpancingo 39016, Mexico; eduardo@navarrozarza.com.mx

**Keywords:** supraclavicular fossa, palpation, haptic ability, ultrasonography, dissection, inferior belly of omohyoid

## Abstract

Background: Palpation, a traditional haptic ability, is used daily by practitioners of all medical and surgical specialties to assess patients. In the current study, one of the authors, in a routine clinical setting, was able to deduce the dynamic features of the putative inferior belly of omohyoid. This led to a proof-of-concept study that yielded results consistent with the clinical findings. Methods: The first part of the study involved a survey of 300 rheumatic disease patients in whom the greater supraclavicular fossa was explored by palpation. While the patient kept the head straight, the clinician placed his middle three fingers 2.5–3 cm dorsal to the clavicle in the window between the sternocleidomastoid and trapezius clavicular insertions, explored the supraclavicular fossa, and palpated the paired contractile inferior belly of the assumed omohyoid during flexion in the three orthogonal planes. In the second part of the study, five normal subjects were examined in a similar manner by the same clinician and had independent ultrasonography performed on the dominant side. Descriptive statistics were used, and Yates’ corrected chi-squared test was applied to certain nominal variables. Additionally, a comparative anterolateral bilateral neck dissection was performed in a cadaveric specimen. Results: Both studies showed that the contractile structure was the inferior belly of omohyoid and that its contraction occurred during anterior neck flexion and was opposite to the side of neck rotation, resembling the sternocleidomastoid. Conclusions: Palpation uncovered a previously unknown function of the inferior belly of omohyoid, suggesting that physical examination of the musculoskeletal system based on palpation may lead to hypotheses worthy of exploration.

## 1. Introduction

The omohyoid consists of a flat, elongated structure with a superior belly attached to the hyoid bone, an intermediate tendon beneath the sternocleidomastoid muscle, and an inferior belly originating from the scapula’s upper border. The inferior belly of omohyoid extends forwards and upwards in the greater supraclavicular fossa, reaching the intermediate tendon. Most of the known functions of omohyoid refer to the superior belly of omohyoid, with textbooks mentioning its steading effect on the hyoid bone as well as its ability to tense the cervical fascia, maintain the neck veins’ patency, and prevent the suction of soft tissues at the lung apices (Table S1) [1,2,3,4,5,6,7,8,9,10,11,12].

The inferior belly of omohyoid has not been extensively researched. Most research has focused on anatomical variations [13,14,15] and their involvement in the omohyoid syndrome. This syndrome occurs when the intermediate tendon lifts the sternocleidomastoid during swallowing, causing transient swelling in the lower neck [16]. It is caused by a failure in the fascial loop that retains the intermediate tendon at its point of inflection, allowing it to bowstring [16,17]. The inferior belly of omohyoid is used as a guide for ultrasonographic blockade of the suprascapular nerve [18], but there are concerns about potential damage to the omohyoid during catheterization of the internal jugular vein [19]. In plastic surgery, it has been successfully used as a functional flap [20]. The inferior belly of omohyoid contracts during resisted contraction at 90 degrees abduction [21]. Other functions of this muscle are assumed and based on its action on the fascia, including maintaining the patency of neck veins and preventing the suction of the soft tissues during prolonged inspiration. The inferior belly of the omohyoid is innervated by the inferior root of the ansa cervicalis [22,23]. Some evidence indicates that it may be innervated by a thin branch of the accessory nerve [24].

In 2011, one of the authors noted a puzzling, bilateral, transversely placed contractile structure while examining the greater supraclavicular fossa of new patients. These unique structures, assumed to be the inferior belly of omohyoid, had peculiar characteristics during certain neck motions. Two observational studies were conducted. The first, based on palpation, aimed to determine the pattern of contraction during the three orthogonal motions of the neck. The second, a proof-of-concept study based on palpation and ultrasonography, aimed to verify that these structures represented the inferior belly of omohyoid.

## 2. Materials and Methods

### 2.1. Study in Rheumatic Disease Patients

In this cross-sectional clinical study, a single rheumatologist (JJC) with over 45 years of clinical experience and a particular interest in the musculoskeletal system retrieved all new patients seen in his private office at the ABC Medical Center in Mexico City, Mexico, from 2011 to 2019. Using the Word 365 search engine from Microsoft Corporation, 800 electronic medical records were identified by searching for the word “omohioides” (omohyoid in Spanish). An interim analysis of the initial 100 patients showed uniformity in their findings, so only 300 consecutive alphabetized records were reviewed. All patients were included in the study, regardless of past or present neck pain or limitations. However, patients with acute neck pain that prevented exploration or those without cervical spine motion (such as those with advanced ankylosing spondylitis or disseminated idiopathic skeletal hyperostosis) would be excluded, although none belonged to the latter category. The sample included 215 females and 85 males, which is in line with the prevalence of rheumatic diseases in Mexico [25], and worldwide [26,27], and all were White predominantly of a European stock. Ninety-one percent were right-handed, 7% were left-handed, and 2% were ambidextrous. Additional information can be found in Table 1. None of the patients had undergone neck surgery.

### 2.2. Clinical Maneuvers

**Figure 1:**
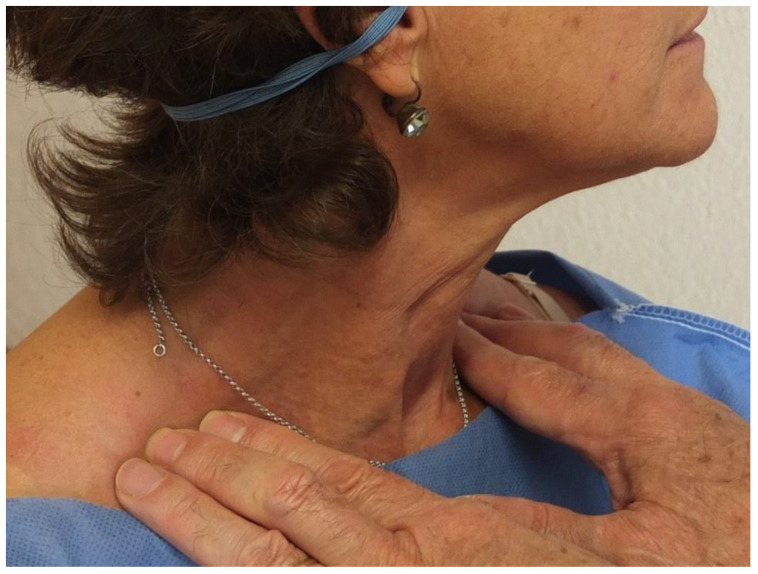
Palpation of omohyoid. With the patient sitting, the examiner placed his index, middle, and ring fingers 2.5 to 3 cm dorsal to the mid-third of the clavicles. The fingers remain in place during the three orthogonal movements of the head.

While the patient was sitting, the examiner explored the larger supraclavicular fossa by palpation and then placed the index, middle, and ring fingers of both hands 2.5 to 3 cm dorsal to the middle third of the clavicles (Figure 1).

During the examination, the median plane was explored by performing flexion and extension movements, the coronal plane bending to the right and left, and the horizontal plane turning the head to the right and left, but only within a pain-free range for the patient. Patients were asked to move smoothly from one extreme to the other without stopping midway. A reproducible transverse hardening beneath the fingers at the examined site at some point during the arc of motion could indicate a contracting inferior belly of omohyoid. Other anatomical structures in the greater supraclavicular fossa in the window between the lateral edge of the clavicular insertion of sternocleidomastoid and the medial border of the clavicular insertion of trapezius that could be confused were considered. These included the subclavian artery, which would exhibit pulsation, and the upper and middle trunks of the brachial plexus, which would feel like nerves without pulsating or contracting, and plucking them would trigger paresthesia. In some cases where there was doubt, patients were asked to tighten their neck skin to distinguish a prominent bundle of platysma from the deep-bound, assumed inferior belly of omohyoid.

### 2.3. Study on Normal Subjects

The cross-sectional study on rheumatic disease patients led to a proof-of-concept study performed on 13 July 2022, at the Hospital Universitario Fundación Jiménez Díaz in Madrid, Spain. The study involved examining five healthy White individuals using palpation (JJC) and ultrasonography (EN, OO-V) with the assistance of two senior anatomists (JMG, JRMV). All participants provided written informed consent and had no current or previous rheumatologic, musculoskeletal, or neurological diseases, neck pain or ailments, or history of neck surgery. The participants consisted of two females and three males, ranging in age from 40 to 62, with a median age of 40. Four of the participants had normal weight, while one was overweight.

### 2.4. Clinical Maneuvers

The patients were explored as those in Mexico City and by the same examiner (JJC).

### 2.5. Ultrasonographic Assessment

Two experts (EN, OO-V), with 27 and 5 years of experience in musculoskeletal ultrasonography, respectively, performed the scanning of the inferior belly of omohyoid with a conventional 2D B-mode real-time scanner (LOGIQ E10, GE Medical Systems Ultrasound and Primary Care Diagnostics, LLC, Wauwatosa, WI, USA) equipped with a multifrequency linear transducer (ML 6–15 MHz). B-mode settings were standardized for the study: B-mode frequency 15 MHz; B-mode gain 50 dB; and dynamic range 63 dB. To identify the inferior belly of omohyoid, with the subjects seated, the transducer was placed transversely and longitudinally 2–3 cm cranial and posterior to the medial end of the clavicle and moved along a downward and posterior slope toward the trapezius on the dominant side. The depth from the skin surface, the transverse diameter, and the thickness of the inferior belly of omohyoid were measured at the mid-clavicle. Additionally, videos were obtained recording the contraction of the inferior belly of omohyoid during neck flexion. The palpatory and ultrasonographic studies were performed independently of each other, supervised by two senior anatomists (JMG and JRMV).

### 2.6. Statistical Analysis

Descriptive results are presented in absolute and relative frequencies, mean ± standard deviations, or mean and standard deviation (SD). Yates’ corrected chi-squared test was used for comparing relative frequencies in some selected nominal variables; in this case, statistical significance was set at 0.05. Data were stored and analyzed using the statistical program SPSS for Windows version 20.0 (IBM Corp., Armonk, NY, USA).

### 2.7. Cadaveric Dissection

To determine if the location of palpation and ultrasonography corresponded with the site of the inferior belly of omohyoid, a study was conducted on a 56-year-old female embalmed corpse. Bilateral omohyoid dissection provided information on the inferior belly of omohyoid relationships with the sternocleidomastoid, the cervical fascia’s middle layer, and the posterior cervical triangle’s neurovascular elements.

### 2.8. Ethical Aspects

The Mexican clinical protocol was approved by the Research Committee of the American British Cowdray (ABC) Medical Center (Mexico City), #ABC-13-08. Since the neck examination was part of the routine two-hour evaluation of all new patients, the committee recommended that signed, informed consent not be required. However, a patient who was photographed gave signed informed consent. The Madrid portion of the study was approved by the Ethics Committee of the Jiménez Díaz Foundation, project 28-06-2022, PIC116-22, and signed informed consents were obtained from all subjects. The anatomical study was performed following the Declaration of Helsinki. The corpse belonged to the Center of Donation of Corpses, Complutense University of Madrid, and was obtained following the donation’s legal procedures.

## 3. Results

### 3.1. Rheumatic Disease Patients

#### Demographic and Palpatory Data (Table 1)

The haptic characteristics and location helped identify the assumed inferior belly of omohyoid in the described area. In most patients, the muscle had a tape-like feeling. However, in some patients, it had unique characteristics. In five patients, the muscle had a sharp posterior border. In four patients, it was thickened and cord-like bilaterally. Three patients had a filiform muscle bilaterally. In two patients with chronic anxiety disorder, the inferior belly of omohyoid felt unusually tight. In one patient with a pronounced dorsal kyphosis, the inferior belly of the omohyoids was still palpable, even submerged behind the clavicles. In the median plane, contraction of the inferior belly of omohyoid was detected in 70.9% of patients from full extension to full flexion, usually bilaterally (ratio 5.6 to 1) (Table 2).

**Figure 2:**
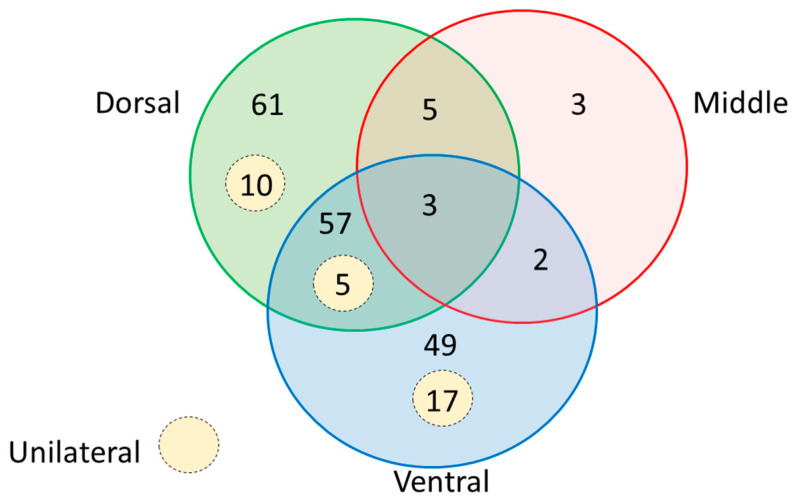
Contraction of the inferior belly of omohyoid in the median plane by thirds of the arc of motion.

A Venn diagram (Figure 2) shows in the median plane, by thirds, where contraction was noted: The dorsal third in 76 patients, the dorsal plus ventral thirds in 65 patients, and the ventral third in 68. Only three patients showed contractions exclusively in the middle third. Furthermore, only three patients showed contractions throughout the arc of flexion. When the contraction was unilateral, it predominated on the right side (ratio 29 to 3). In only 2.4% of patients, a contraction was noted in the opposite motion, i.e., from full flexion to full extension, at the beginning of extension.

In the coronal plane (Table 2), contractions were recorded on either side in 2.0% of patients. In the horizontal plane, contralateral contractions were felt in 117/298 (39.2%) patients, compared to 68/298 (22.8%) ipsilateral or bilateral contractions (*p* = 0.0002). The participation of the contralateral inferior belly of omohyoid during horizontal movements became more evident when ipsilateral contractions, 41/298 (13.7%), were compared to contralateral contractions or both, 144/298 (48.3%) patients (*p* < 0.000001). Age, sex, and body mass index did not affect the inferior belly of omohyoid contraction.

### 3.2. Normal Subjects

#### 3.2.1. Palpation of IOH in Active Neck Motion

Palpatory contraction of the inferior belly of omohyoid was perceived during neck flexion in all five subjects, during extension in none, opposite to the side of head rotation in three, and ipsilateral to the side of rotation in none.

#### 3.2.2. Ultrasonographic Findings

**Figure 3:**
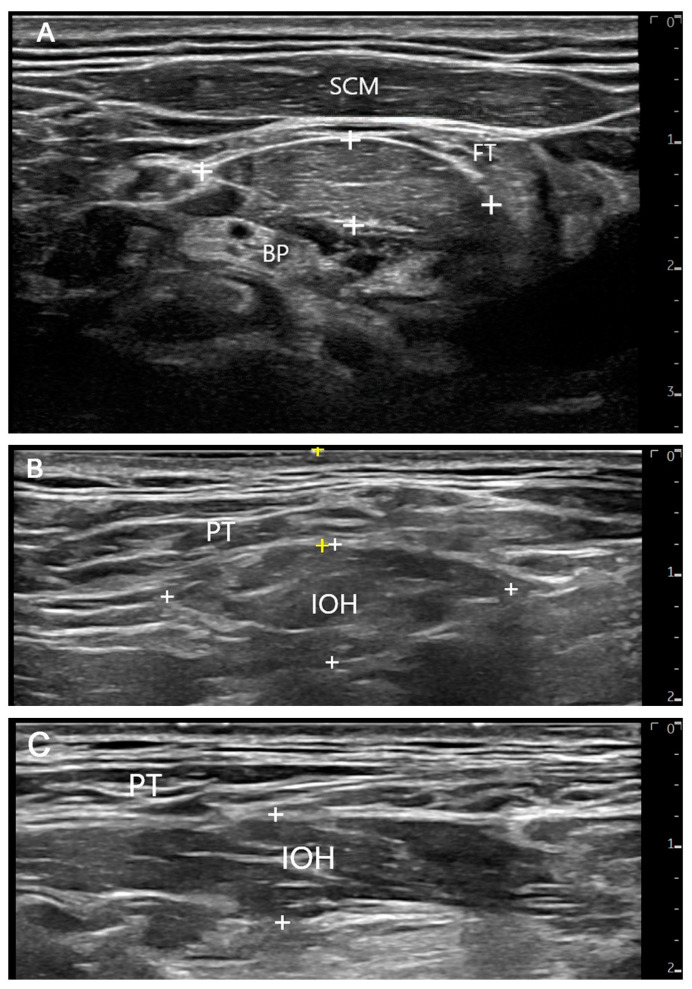
Ultrasound of inferior belly of omohyoid. (**A**) The inferior belly of omohyoid (IOH) between crosses in short axis (transverse), close to the intermediate tendon of omohyoid and beneath sternocleidomastoid (SCM) (FT, fat tissue; BP, brachial plexus). (**B**) Short-axis scan of the IOH (between white crosses) and distance between the IOH and the skin (between yellow crosses) at mid-clavicle. (**C**) Long-axis (longitudinal) scan of the IOH (between white crosses). PT, platysma. The scale bar indicating centimeters is shown on the right side of the ultrasound images.

In the five subjects, the inferior belly of omohyoid was identified in a transverse scan with a rounded-ovoid shape and a longitudinal scan with a ribbon shape (Figure 3). Using the mid-clavicle as a reference, the mean (SD) distance from the skin to the inferior belly of omohyoid was 7.19 (2.14) mm (range 6.53 to 11.85 mm). The transverse diameter of IOH had a mean (SD) of 13.72 (4.42) mm (range 10.53 to 19.81 mm), and the thickness had a mean (SD) of 8.5 (1.48) mm (range 5.7 to 9.66 mm). During flexion, the inferior belly of omohyoid contracted in four of the five subjects. An example of contraction is shown in Video S1.

#### 3.2.3. Anatomical Findings

**Figure 4:**
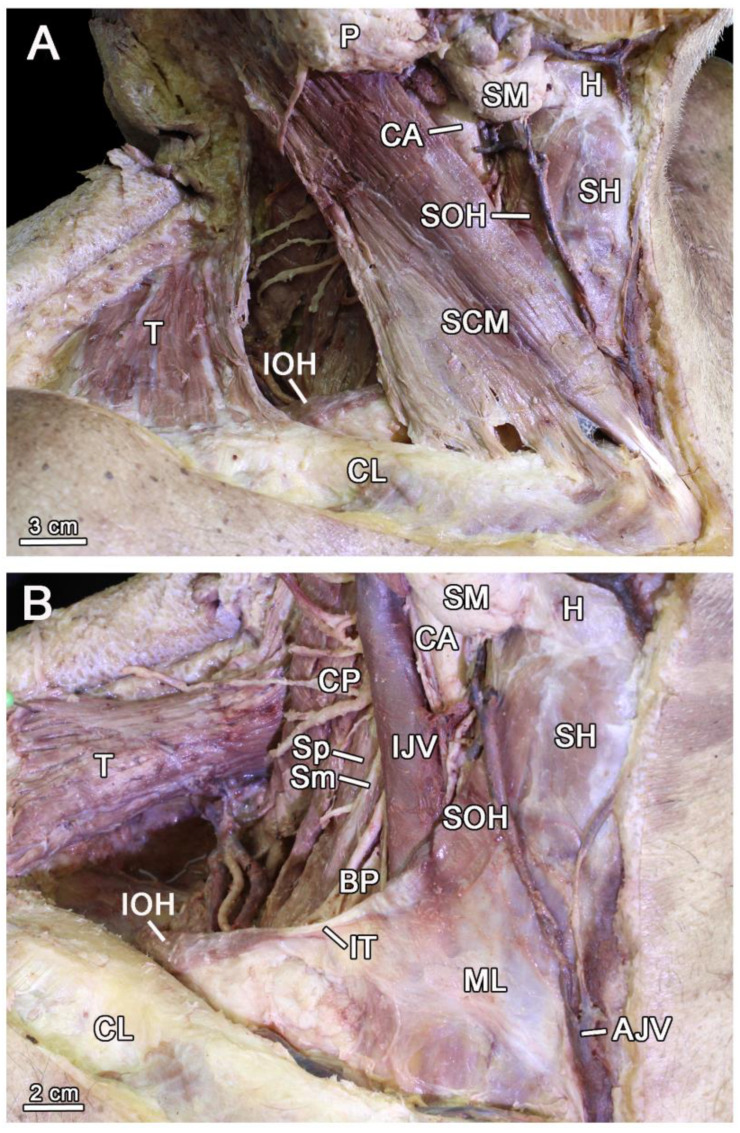
Dissection of the posterior triangle of the neck. (**A**) Lateral view of the dissected posterior triangle of the neck. (**B**) Anterolateral view of the dissected right neck. Trapezius and the clavicular and sternal portions of SCM were disinserted and flipped. AJV, anterior jugular vein; BP, brachial plexus; CA, common carotid artery; CL, clavicle; CP, cervical plexus; H, hyoid bone; IJV, internal jugular vein; IOH, inferior belly of omohyoid; IT, intermediate tendon of omohyoid; ML, middle layer of the cervical fascia; P, parotid gland; SCM, sternocleidomastoid; SH, sternohyoid; Sm; scalenus medius. SM, submandibular gland; SOH, the superior belly of omohyoid; Sp, scalenus posterior; T, trapezius.

Upon dissection of the anterolateral neck, the inferior belly of omohyoid was exposed in the posterior cervical triangle at the site where the inferior belly of omohyoid palpations were performed. Once the descending portion of trapezius and the clavicular and sternal pieces of sternocleidomastoid were disinserted and flipped over, the origin of the inferior belly of omohyoid in the superior border of the scapula, its intermediate tendon, and the superior belly of omohyoid insertion in the hyoid were all seen. The direction of the inferior belly of omohyoid, from the scapular insertion, was inward, upward, and forward. The middle layer of the cervical fascia extended from one omohyoid to the other. The intermediate tendon was immediately anterior to the internal jugular vein, and the inferior belly of omohyoid rested on the scaleni and elements of the brachial plexus (Figure 4).

## 4. Discussion

To our knowledge, this is the first comprehensive analysis of the paired inferior belly of omohyoid through palpation while performing neck movement in the three orthogonal planes. The observed phenomena were a result of the muscle’s perceived haptic properties and the impact of the contracting muscle on the explorer’s fingers. With basic knowledge of the neck’s lower lateral triangle anatomy, we could recognize, identify, and interact with a muscle buried in the greater supraclavicular fossa. The primary finding in the first part of this study was the contraction pattern of the assumed inferior belly of omohyoid (Table 2). In the median plane, the inferior belly of omohyoid contracted during flexion from an extended neck position, and in the horizontal plane, predominantly contralateral to the side of rotation. Findings in the coronal plane were neutral. Similar results were observed in the Madrid small proof-of-concept study that confirmed that the assumed inferior belly of omohyoid was indeed the inferior belly of omohyoid. This contraction pattern of the inferior belly of omohyoid resembled sternocleidomastoid [28], suggesting that the inferior belly of omohyoid and sternocleidomastoid are agonists in their action on the neck. The innervation of the inferior belly of omohyoid from the ansa cervicalis (C1, C2, and C3) and of sternocleidomastoid from the spinal nerve and the ventral rami of the second, third, and sometimes fourth cervical spinal nerves [7,22,23] could support these results. There is an unconfirmed study in which, in two patients with implanted electrodes in the superior belly of omohyoid and the inferior belly of omohyoid, the accessory nerve provided the primary motor innervation of the inferior belly of omohyoid [24]. Interestingly, in a patient with congenital torticollis due to a fibrotic cord replacing the omohyoid [29], the torticollis observed was like congenital torticollis due to a shortening of sternocleidomastoid. The difference was that, on X-rays, the trachea was pulled toward the fibrotic side in the case of omohyoid fibrosis due to its hyoid insertion. In addition to the rheumatic disease, some study patients had concurrent neurological conditions that highlighted the inferior belly of omohyoid innervation. For example, a 62-year-old woman had a stiff person syndrome with high titers of anti-glutamic acid decarboxylase (GAD) antibodies. When she opened her mouth, the sternocleidomastoid and the inferior belly of omohyoid contracted on both sides of her neck. A 69-year-old woman had rheumatoid arthritis and right hemifacial spasms. The face muscles (C7) and platysma (C7) contracted with the spasms, but the inferior belly of omohyoid only contracted with neck flexion. Finally, a 45-year-old woman had right hemifacial atrophy with a loss of subcutaneous fat on the right side of her face and the right posterior neck triangle. Although the right inferior belly of omohyoid was prominent, muscle volume appeared equal on both sides of her neck. Why the inferior belly of omohyoid was not palpable in some subjects could relate to a short neck, excessive supraclavicular fat, anatomic variation, or a failure to detect the contraction.

One limitation of this study is the potential for observer bias, as the same observer examined the patients in Mexico City and the normal subjects in Madrid. However, the similarity of the clinical and ultrasonographic findings in the latter study suggests that the impact of this bias was likely minimal. Additionally, the inclusion of patients with diverse rheumatic diseases in the Mexico study may not be a drawback, as medical and surgical specialty learners and practitioners often face similar challenges. The protocol did not include scapular elevation, which could have provided further insight into the findings. Furthermore, the assessment of neck length was deemed too subjective and was ultimately discontinued. However, in hindsight, this decision may have been incorrect, given the results. Finally, the Madrid proof-of-concept study included a small number of normal subjects, which were a convenience sample. This study confirmed that the structure identified as the inferior belly of the omohyoid in the 300-patient study was indeed the inferior belly of omohyoid. The literature addressing the inferior belly of omohyoid innervation from a functional standpoint is surprisingly meager. For example, J.V. Basmajian complained in 1967 that no electromyography (EMG) studies had addressed the infrahyoid musculature [30]. Since then, in addition to the Vanneuville et al. study [22], there has been an electromyographic study of the superior belly of omohyoid but not the inferior belly of omohyoid, in which isometric contractions were noted on neck flexion and unspecified rotation [31], and the Palmerud et al. study [32], cited by Toledano and Dar [21], in which omohyoid activity was greatest in 60 and 90 degrees abduction in the scapular plane. 

The study’s findings invite speculation. Based on palpation and ultrasonography, the inferior belly of omohyoid contracts similarly to the sternocleidomastoid. Unlike a strong sternocleidomastoid, which attaches firmly to the sternum and the medial end of the clavicle, the inferior belly of omohyoid attachment to the upper border of the scapula is off-center and weak. In humans, an upward rotation of the scapula (counterclockwise if one sees the right scapula from behind and the opposite movement if one sees the left scapula) is necessary to lift the arm [33]. This action is primarily achieved by the lower digitations of serratus anterior pulling on the inferior angle of the scapula and the upper trapezium pulling upward and inward on the lateral clavicle and acromion. This motion probably tenses the omohyoid’s inferior belly, and this muscle’s proprioceptor input could be a sensor of scapular rotation around its AP axis [32]. The observations of Toledano and Dar [21] of a synergy between the shoulder abductors and the lower belly of omohyoid during resisted shoulder abduction with the arm abducted 90 degrees, which also implies upward rotation of the scapula, are of particular interest, as the contraction of a muscle that links the scapula to, indirectly, the cervical spine may be triggered at either end. Further surface electromyography [32,34] at the upper, mid, and low trapezius, inferior belly of omohyoid at the described palpation window, sternocleidomastoid, deltoid, and supraspinatus during cervical and shoulder motions could shed light on this complex muscle coordination. Moreover, a study of the inferior belly of omohyoid proprioceptors in different portions of the muscle would be of great interest [35]. Finally, based on the above observations, the authors believe that basic clinical skills and ultrasonography have great untapped potential in musculoskeletal research. Palpation is useful for identifying superficial ligaments, tendons, and nerves, such as the infrapatellar branch of the saphenous nerve. This potential for palpation was demonstrated by the dynamic palpation of the lateral bands of the extensor apparatus of the fingers [36]. The authors have ongoing projects based on palpation of forearm tendons, muscles, and hand ligaments. Although some could consider it a step back in anatomical research, this method may represent a significant step forward in linking clinical medicine with anatomy.

## 5. Conclusions

According to this study, palpation of the greater supraclavicular fossa during neck motion showed that the contraction pattern of the inferior belly of omohyoid is like that of the sternocleidomastoid. This was later confirmed by ultrasonography.

## Figures and Tables

**Table 1:** Demographic and clinical data from 300 rheumatologic patients.

	*N*	Mean ± S.D.	Range
Age	300	56.2 ± 15.3	16–89
Height (cm)	298	163.1 ± 8.7	142.5–189.0
Weight (kg)	300	66.8 ± 14	26.6–169.0
Body mass index	298	25.7 ± 12.0	16.3–49.4
Gender	300	Female 215	Male 85
Handedness	287	Right 261	
		Left 19	
		Ambidextrous 7	
Neck type	143	Short 31	
		Medium 63	
		Long 49	

The primary diagnoses of the patients appear in Table S2.

**Table 2:** Palpatory contraction of the inferior belly of the omohyoid during neck motion in three orthogonal planes.

Median Plane (*N* = 299 ^1^)	Side of contraction	
From maximal extension to flexion		212 (71.0%)
	Bilateral	180 (60.2%)
	Unilateral, right side	29 (9.7%)
	Unilateral, left side	3 (1.0%)
From flexion to maximal extension		7 (2.0%)
Coronal plane (*N* = 298 ^1^)	Side of contraction	
Right flexion	Right	6 (2.0%)
	Left	8 (2.7%)
	Both	5 (1.7%)
Left flexion	Right	8 (2.7%)
	Left	12 (4.0%)
	Both	5 (1.7%)
Horizontal plane (*N* = 298 ^1^)	Side of contraction	
Right turn	Right	23 (7.7%)
	Left	57 (19.1%)
	Both	18 (6.0%)
Left turn	Right	60 (20.2%)
	Left	18 (6.0%)
	Both	9 (3.0%)

^1^ Difference with 300 due to missing data.

## Data Availability

Data presented in this study are available on request due to privacy restrictions from the corresponding author.

## Supplementary Materials

The following supporting information can be downloaded at https://www.mdpi.com/article/10.3390/diagnostics13183004/s1, Table S1: described actions and innervation of omohyoid in Anatomy Textbooks; Table S2: diagnoses in 300 rheumatologic patients; Video S1: In this video, obtained during neck flexion, the transducer was transversely placed (short axis) over the middle third of the supraclavicular fossa. Skin and subcutaneous/fatty tissue appear on the top. Platysma fibers cannot be identified within this tissue, probably due to the orientation of its fibers. The elongated muscle on the top right is the clavicular head of sternocleidomastoid. The rounded muscle, part beneath sternocleidomastoid, and part beneath the subcutaneous tissue/fat is the inferior belly of omohyoid (IOH). This muscle is seen to contract at the beginning of neck flexion. The “empty” dark area beneath IOH is the subclavian vein, and the stronger signal beneath this vein is the subclavian artery. This video shows, by default, the scale bar used during the study.
