# Supplementary material for: Palpation and Ultrasonography Reveal an Ignored Function of the Inferior Belly of Omohyoid: A Case Series and a Proof-of-Concept Study
Source: Diagnostics (Basel). 2023 Sep 20;13(18):3004. doi: 10.3390/diagnostics13183004 (PMC10529686; doi:10.3390/diagnostics13183004)
Supplement: Supplementary file 1 [file diagnostics-13-03004-s001.zip › Table S1.pdf]

Table S1. Described actions and innervation of omohyoid in Anatomy Textbooks

Year	Author	Ed.	Action on			Innervation
			**Hyoid bone**	**Fascia**	**Veins; other**	
1901	Poirier, Charpy	2nd	Stabilizes	Tenses	Maintains vein patency[1]	Ansa cervicalis
1923	Spalteholz	7th	Pulls down	Tenses	NM*	Ansa cervicalis
1948	Testut, Latarjet	9th	Pulls down and back	NM	No effect	Ansa cervicalis
1959	Orts Llorca	2nd	Pulls down	Tenses	Maintains vein patency*	Ansa cervicalis
1969	Lockhart, Hamilton, Fyfe	2nd	Pulls down	NM	NM	Ansa cervicalis
1975	Basmajian (Grant´s)	9th	Pulls down	NM	NM	Ansa cervicalis
1981	Romanes	12th	Pulls down	NM	NM	Ansa cervicalis
1986	O'Rahilly, Müller	5th	Pulls down	Tenses	Maintains vein patency; relieves pressure on lung apices*	Ansa cervicalis
1999	Rouvière, Delmas	10th	Pulls down and back	NM	NM	Ansa cervicalis
2005	Schuenke, Schulte, Schumacher		Pulls down	Tenses	Maintains vein patency*	Ansa cervicalis
2021	Standring	42nd	Pulls down	Tenses	It prevents suction inwards for the soft parts*	Ansa cervicalis
2023	Dalley, Agur	9th	Pulls down, retracts and steadies hyoid	NM	NM	Ansa cervicalis

[1]Possible actions involving the inferior belly of omohyoid

NM= not mentioned
