# Supplementary material for: Palpation and Ultrasonography Reveal an Ignored Function of the Inferior Belly of Omohyoid: A Case Series and a Proof-of-Concept Study
Source: Diagnostics (Basel). 2023 Sep 20;13(18):3004. doi: 10.3390/diagnostics13183004 (PMC10529686; doi:10.3390/diagnostics13183004)
Supplement: Supplementary file 1 [file diagnostics-13-03004-s001.zip › Table S2.pdf]

Table S2. Diagnoses in 300 rheumatologic patients.

**Diagnoses**	**N**	**%**
Osteoarthritis	44	14.7
Rheumatoid arthritis	39	13.0
Primary Sjögren´s syndrome	27	9.0
Fibromyalgia	18	6.0
Gout	11	3.7
Systemic lupus erythematosus	9	3.0
Polymyalgia rheumatica	8	2.6
Scleroderma	6	2.0
Other diagnoses	138	46.0
